# CT Diagnosis of Tibial Post Fracture in a Posterior-Stabilized Total Knee Arthroplasty

**DOI:** 10.5334/jbsr.4202

**Published:** 2026-02-02

**Authors:** Alexander Cotza, Filip Robijns, Jan Vandevenne

**Affiliations:** 1 Department of Radiology, Ziekenhuis Oost-Limburg, Genk, Belgium; 2 Department of Orthopaedics, Ziekenhuis Oost-Limburg, Belgium; 3 Faculty of Medical and Life Sciences, Hasselt University, Hasselt, Belgium

**Keywords:** total knee arthroplasty, posterior-stabilized knee prosthesis, tibial post fracture, polyethylene insert, computed tomography, Hounsfield units

## Abstract

Fracture of the polyethylene tibial post is a rare complication of posterior-stabilized total knee arthroplasty and is frequently missed on conventional radiography due to the radiolucent nature of polyethylene. We report a case of post-traumatic instability in which computed tomography demonstrated a displaced polyethylene fragment with characteristic low attenuation values. The diagnosis was confirmed intraoperatively, and isolated polyethylene exchange resulted in a good clinical outcome.

*Teaching point:* CT attenuation measurements can aid in identifying displaced polyethylene fragments and facilitate the diagnosis of tibial post fracture in posterior-stabilized knee arthroplasty.

## Introduction

Posterior-stabilized total knee arthroplasty (PS-TKA) relies on a cam–post mechanism to substitute for the posterior cruciate ligament and provide posterior stability during knee flexion [1]. Fracture of the polyethylene tibial post is an uncommon but recognized complication and may result in acute instability, pain, mechanical symptoms, and joint effusion [2, 3].

Diagnosis is challenging, as polyethylene is radiolucent on conventional radiographs and radiographic findings may be subtle or overlooked [1, 4]. Arthroscopy, MRI, and CT arthrography have been described as diagnostic tools, whereas the role of standard CT with attenuation analysis remains underreported [1]. We present a case in which CT with Hounsfield unit (HU) measurements enabled the identification of a displaced polyethylene fragment following trauma.

## Case Report

A male patient with a PS-TKA of the left knee presented with new-onset instability nine years after implantation. Symptoms developed after a traffic accident. Before the trauma, the patient reported satisfactory prosthetic function, although intermittent joint effusions had occurred.

After the accident, he complained of subjective instability, recurrent hydrops, and an audible clicking sensation. Orthopedic evaluation raised suspicion of mechanical failure of the prosthesis.

During clinical stability testing at orthopedic consultation, the absence of a posterior stop was noted, indicating failure of the cam–post mechanism. Joint aspiration yielded approximately 50 mL of clear, mildly hemorrhagic synovial fluid. Microbiological cultures were negative, excluding infection.

**Figure 1:**
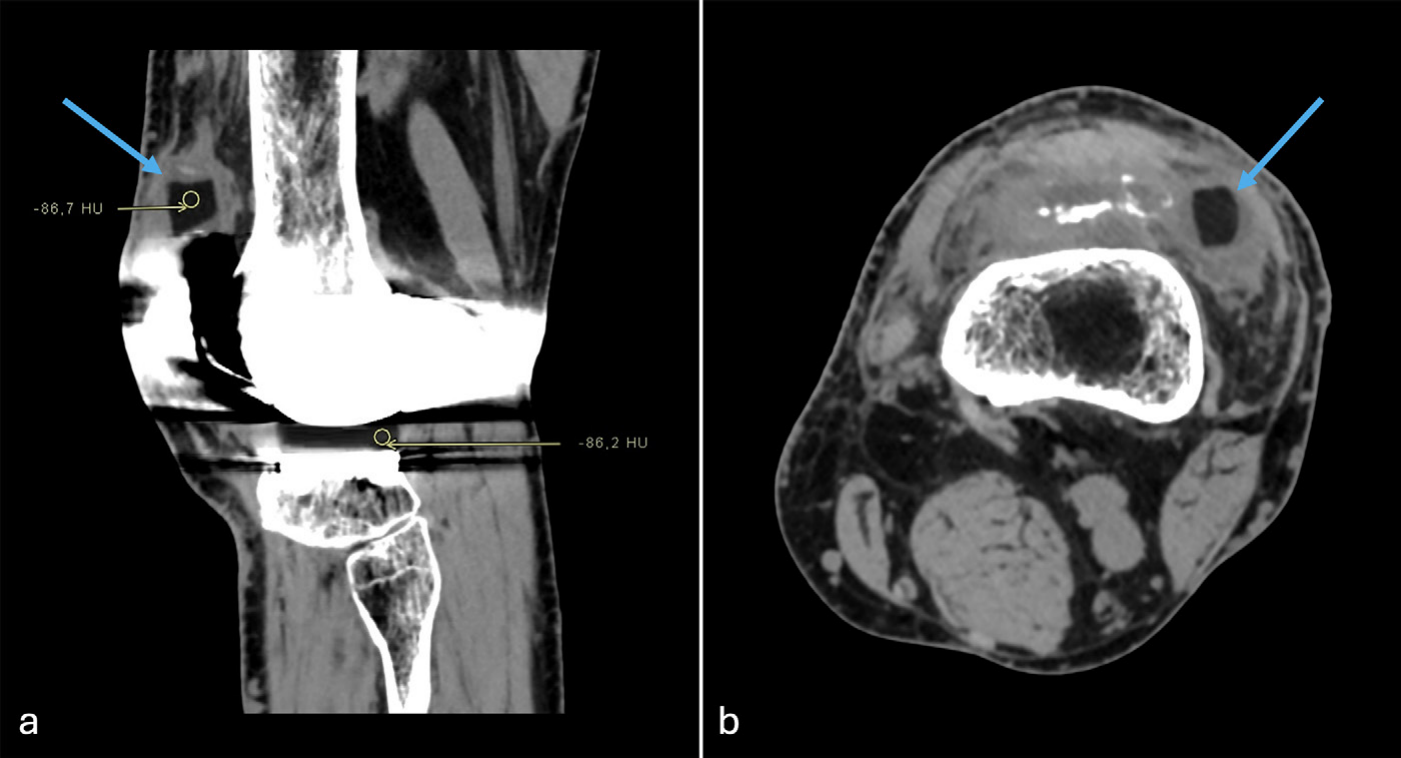
CT images displayed in a soft-tissue window demonstrating a displaced polyethylene tibial post fragment (blue arrows). **(a)** Sagittal image showing region-of-interest measurements within the displaced fragment and the intact tibial insert, revealing similar low attenuation values (approximately −86 HU), consistent with polyethylene. **(b)** Axial image demonstrating the well-defined displaced fragment in the lateral suprapatellar region.

CT of the left knee was performed. Following targeted evaluation prompted by clinical findings, CT demonstrated a well-defined cylindrical structure located in the lateral recess of the suprapatellar bursa (Figure 1). This structure showed homogeneous low attenuation values of approximately −86 HU, identical to those measured in the intact polyethylene tibial insert. No fracture of metallic components or periprosthetic bone injury was identified. These findings were consistent with a displaced polyethylene fragment, suggestive of fracture of the tibial post.

**Figure 2:**
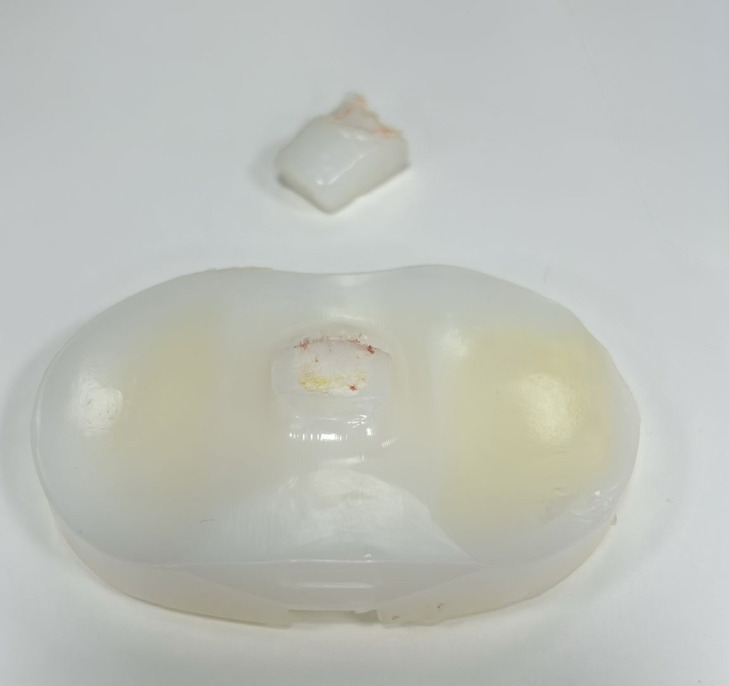
Retrieved polyethylene tibial insert demonstrating fracture of the tibial post, which was confirmed intraoperatively during revision surgery.

Revision surgery was subsequently performed. Intraoperatively, fracture of the polyethylene tibial post was confirmed (Figure 2). The fractured fragment was retrieved from the superolateral suprapatellar region, corresponding to the CT findings. An isolated polyethylene insert exchange was performed. Postoperatively, the patient reported marked improvement in stability, with progressive recovery of range of motion.

## Discussion

Fracture of the polyethylene tibial post is a rare but clinically significant complication of PS-TKA [2–4]. Clinical presentation is often nonspecific and may include pain, instability, recurrent effusion, clicking, or locking [3, 5]. Trauma, hyperextension, and repetitive impingement of the cam–post mechanism have been proposed as contributing factors [2, 3].

Radiological diagnosis is challenging. Polyethylene components are radiolucent on conventional radiographs, and fractures may be overlooked or misinterpreted [1, 4]. Previous reports have emphasized arthroscopy, MRI, or CT arthrography for diagnosis of tibial post fractures [1]. Hsu et al. demonstrated that CT arthrography can outline fractured polyethylene fragments by contrast delineation [1].

In the present case, standard CT without intra-articular contrast was sufficient to identify the displaced fragment. Measurement of attenuation values was instrumental in characterizing the lesion as polyethylene, as the HU values matched those of the intact insert.

Early diagnosis is essential, as delayed recognition may lead to progressive damage to surrounding soft tissues or metallic components [4]. When other prosthetic components remain well fixed, isolated polyethylene exchange represents an effective treatment option [1, 5].

## Conclusion

Fracture of the polyethylene tibial post should be considered in patients with PS-TKA presenting with post-traumatic instability and recurrent effusion. Standard CT, combined with careful image review and HU analysis, can identify displaced polyethylene fragments and facilitate timely diagnosis.
